# A break in parental interaction does not affect the temporal dependency of infant social engagement, but disrupts non-social engagement

**DOI:** 10.1038/s41598-018-33270-9

**Published:** 2018-10-11

**Authors:** Whitney I. Mattson, Daniel S. Messinger, Devon N. Gangi, Nicholas D. Myers

**Affiliations:** 1 Center for Biobehavioral Health, The Research Institute at Nationwide Children’s Hospital, Columbus, Ohio United States of America; 2 Department of Psychology, University of Miami, Coral Gables, Florida United States of America; 3 Department of Pediatrics, University of Miami, Coral Gables, Florida United States of America; 4 Department of Music Engineering, University of Miami, Coral Gables, Florida United States of America; 5 Department of Electrical & Computer Engineering, University of Miami, Coral Gables, Florida United States of America; 6 MIND Institute, University of California, Davis, Sacramento, California United States of America; 7 Department of Counseling, Educational Psychology and Special Education, Michigan State University, East Lansing, Michigan United States of America; 8 Department of Kinesiology, Michigan State University, East Lansing, Michigan United States of America

## Abstract

Infant looking patterns during interaction offer an early window into social and nonsocial engagement. Recent evidence indicates that infant looks exhibit temporal dependency—one look duration predicts the next look duration. It is unknown, however, whether temporal dependency emerges as infants structure their own looking or whether it is influenced by interaction. We examined whether a perturbation of social interaction affected temporal dependency. Using the Face-to-Face/Still-Face procedure, we compared temporal dependency during parental interaction (the Face-to-Face & Reunion episodes) to parental non-responsiveness (the Still-Face episode). Overall, the durations of successive infant looks were predictable; past behavior constrained current behavior. The duration of one look at the parent (*Face Look*) predicted the duration of the next *Face Look*. Likewise, the duration of a look at any place that was not the parent’s face (*Away Look*) predicted the duration of the next *Away Look*. The temporal dependency of *Face Looks* (social engagement) was unaffected by the Still-Face perturbation, but the temporal dependency of *Away Looks* (nonsocial engagement) declined during the Still-Face. Infant temporal structuring of engagement during social looking is not dependent on parental interaction while the disruption of interaction affects infants’ structuring of their own non-social engagement.

## Introduction

**Figure 1:**
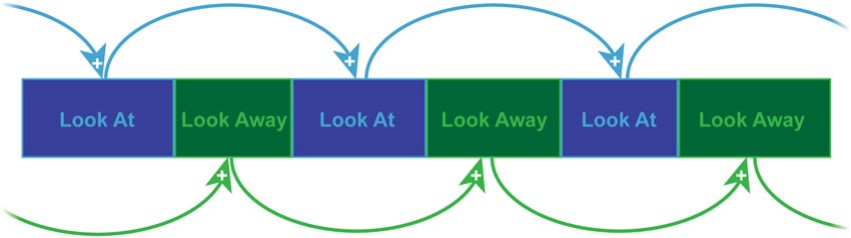
An illustration of the temporal dependency model. The durations of successive *Face Looks* are predicted by the duration of the previous *Face Look*. The durations of successive *Away Looks* are predicted by the duration of the previous *Away Look* from that same target. One previous duration prediction is illustrated.

Infant looking is used to investigate early engagement and attention in procedures such as the Face-to-Face/Still-Face (FFSF)^1,2^. Temporal dependency refers to a positive association between the durations of consecutive behaviors (e.g., longer behaviors are followed by longer behaviors) (see Fig. 1)^3,4^. While the temporal dependency of looking behavior has been documented in face-to-face infant-parent interaction^5^, the source of that temporal dependency is not fully understood. Temporal dependency may reflect infants’ active structuring of their own looking over time or the influence of their interactive partners. The current study investigates whether temporal dependency declines in strength when parents are non-responsive and not engaging in interaction.

## Looking Measures

Infant looking is often parameterized as the total time an infant spends looking at the parent’s face^6–10^. However, this approach ignores individual infant looks and their associations in time. These types of associations in time have proven a productive area of analysis in a variety of biological processes^11^. Early researchers suggested that associations between successive look durations (comprised of a paired look at and a look away from the parent) were stochastic^12^. By contrast, a dynamic systems perspective focuses on the prediction of sequences of individual actions in context^13^. Informed by this dynamic systems approach, we investigated the possibility of non-random variability in looking behavior.

## Related Approaches to Looking Behavior

Although a distinct construct, temporal dependency is similar to the auto-correlation component of a time-series analysis, which may index self-regulatory processes^14^. There is, however, a crucial distinction. Auto-correlation refers to the association between infant behavior measured in fixed, predetermined intervals (e.g., is behavior at second *t* predicted by the previous second of behavior, *t-1*)^15,16^. By contrast, temporal dependency involves associations between the durations of consecutive *events* such as looks at a target (see Fig. 1)^3^, which are determined by the infant’s actions, rather than on fixed units of time. While both types of analysis focus on sequences, a temporal dependency approach is especially relevant to analyses of behaviors parameterized dichotomously (e.g., looking at and away from a social partner).

## Temporal Dependency

A recent report^5^ indicated that the durations of successive infant looks *at* the parent’s face during a face-to-face interaction positively predicted the duration of the next look at the parent. That is, longer looks tended to follow longer looks, and shorter looks tended to follow shorter looks. However, it was not clear whether this temporal dependency effect was due to the infants’ self-organization of their own looking behavior or to the scaffolding influence of interaction with the parent. Further, this report^5^ used a relatively small sample (*n* = 13) and predicted the durations of looks at and away from the parent without centering these variables within infant, which may confound within-individual and between-individual sources of variation^17^. The current study addresses these gaps by employing a larger sample, centering within individual, and addressing the effects of parental interaction in an experimental protocol.

## Look Types and Temporal Dependency

The temporal dependency of *Face Look* (a measure of social engagement) and *Away Look* (a measure of non-social engagement) are key components of social interaction dynamics. Measures of looking during social interaction tend to capture gazes at the partner. However, crucial information may also be contained in the gazes away from a social partner. In a recent report there was temporal dependency in both *Face Looks* and *Away Looks* during interaction^5^. The previous look at the parent (*Face Look)* predicted the current *Face Look* duration. The duration of the previous look away from the parent (*Away Look*) predicted the current *Away Look* duration. There was no evidence that these looks directly influenced the duration of the other look type^5^.

## The Face-to-Face/Still-Face Procedure (FFSF)

The FFSF protocol involves an episode of parent-infant interaction (Face-to-Face) followed by an episode of the parent not responding to the infant (Still-Face) and then resumption of interaction (Reunion)^1^. During the Still-Face, infants show decreases in gaze to the parent and positive affect and more negative affect than in the Face-to-Face episode, followed by partial recovery in the Reunion episode^18^. This still-face effect is present from the age of one month and is fully developed by six months of age^7,19^. In addition to low-risk, typically developing infants, still-face effects are evident in risk populations, such as infants who are preterm, at familial risk for autism spectrum disorder, or experienced prenatal substance exposure e.g.^20–22^. Infant behavior during the FFSF has been linked to later attachment quality and behavior problems^2^. This procedure provides an excellent structure in which to examine the potential influence of interaction on infants’ temporal dependency due to the built-in contrast between interactive (Face-to-Face and Reunion) and non-interactive (Still-Face) episodes. An absence of differences in temporal dependency between the Still-Face and the Face-to-Face/Reunion episodes would suggest that temporal dependency is an internal infant-driven process. By contrast, if temporal dependency differs between the Still-Face and Face-to-Face/Reunion, it might suggest that interaction facilitates the structuring of infant behavior in time.

## Hypotheses

We examined three central questions: (1) is the temporal dependency of *Face Looks* affected by the Still-Face; (2) is the temporal dependency of *Away Looks* affected by the Still-Face; and (3) are the overall levels of infants’ temporal dependency in *Face Looks* and *Away Looks* related, independent of their association at the level of looks? We hypothesized that both (1) Face Looks and (2) Away Looks would be disrupted in the Still-Face. Finally, we hypothesized (3) that that an individual infants’ *mean* level of temporal dependency for Face Looks and *Away Looks* would be associated, while the durations of adjacent *individual Face Looks* and *Away Looks* would not be associated.

## Results

### Description of Looks

The number and duration of individual *Face Looks* and *Away Looks* in the FFSF were examined. See Table 1 for a summary of these characteristics. Overall, look durations followed a lognormal distribution and all durations and counts were log-transformed (log10[x + 1]) prior to analyses^5,23^.

**Table 1:** Characteristics of *Away Look* and *Face Look*.

Look Type		Mean Duration in Seconds (*SD*)	Mean Number of Looks (*SD*)
*Overall*			
*Face Look*		3.21 (*4.66*)	29.94 (*21.82*)
*Away Look*		5.90 (*10.11*)	30.28 (*21.72*)
*By Episode*			
Face-To-Face	*Face Look*	3.08 (*4.47*)	13.66 (*12.17*)
	*Away Look*	4.61 (*8.09*)	14.05 (*10.35*)
Still-Face	*Face Look*	2.77 (*3.68*)	7.13 (*5.64*)
	*Away Look*	9.26 (*14.02*)	28.11 (*16.18*)
Reunion	*Face Look*	3.57 (*5.24*)	10.72 (*7.81*)
	*Away Look*	5.56 (*9.26*)	45.95 (*22.51*)

#### Face Looks

The final model for predicting look durations at the parent was one in which two previous look durations (*D*_*n−1 ij*_ and *D*_*n−*2 *ij*_) and the intercept of *Face Looks* (*π*_*0i*_) predicted the next look duration (*Y*_*ij*_), as described in this simplified model of level 1 effects:

$${Y}_{ij}={\pi }_{0i}+{\pi }_{1i}{D}_{n-1ij}+{\pi }_{2i}{D}_{n-2ij}+{\varepsilon }_{ij}$$

**Figure 2:**
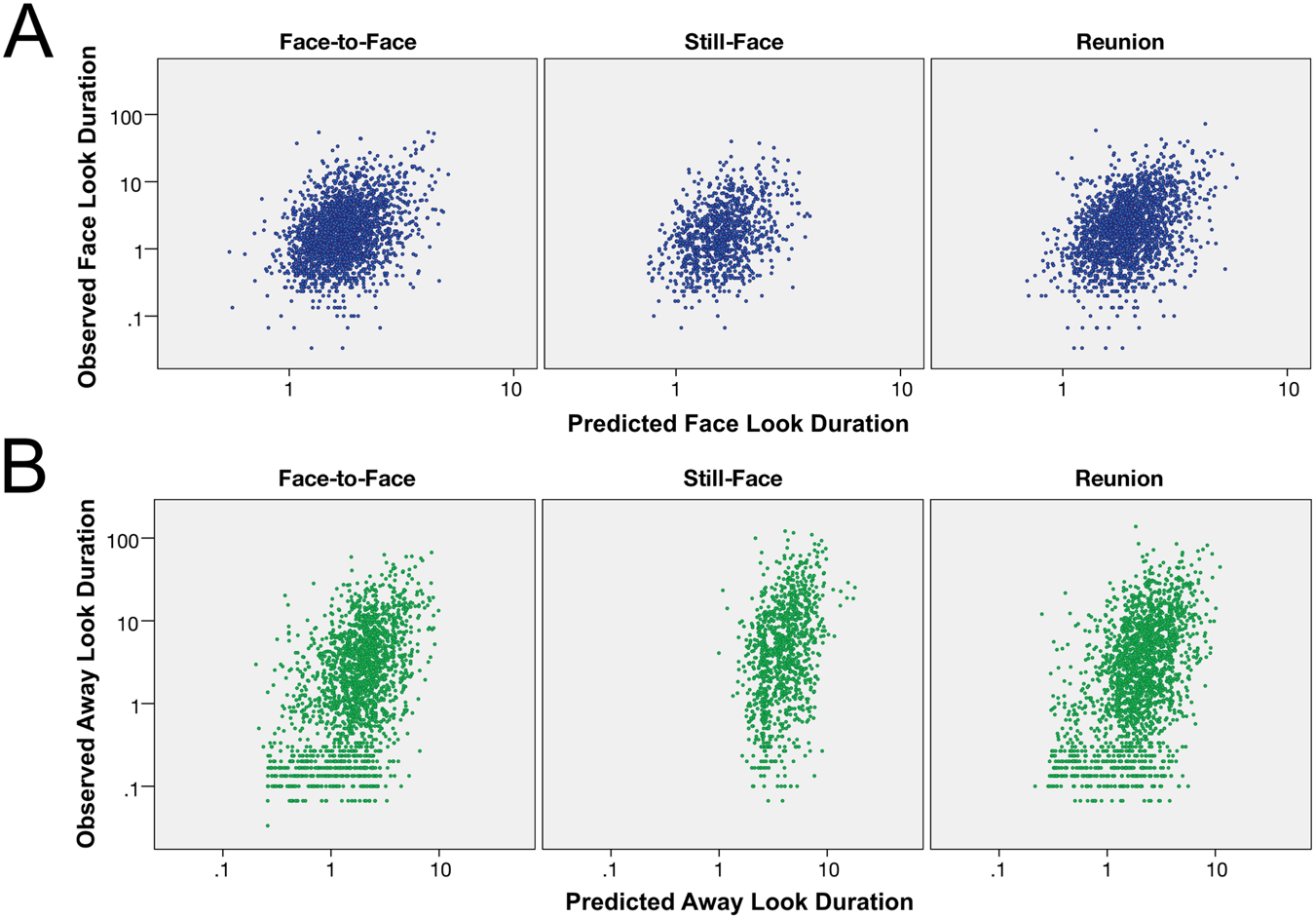
(**A**) The relationship of observed *Face Look* durations and those predicted by the final model. The final model included terms for the *Face Look* duration one previous and the *Face Look* duration two previous. X and Y-axes are not identical. Predicted *Face Look* durations ranged from 0.54 s to 5.96 s. Observed *Face Look* durations ranged from 0.03 s to 100 s. (**B**) The relationship of observed *Away Look* durations and those predicted by the final model. The final model included terms for the *Away Look* duration one previous, the Still-Face vs. Face-to-Face and Reunion, and the interaction of these terms. X and Y-axes are not identical. Predicted *Away Look* durations ranged from 0.20 s to 18.03 s. Observed *Away Look* durations ranged from 0.03 s to 137.72 s.

Specifically, one previous *Face Look* (lag 1) predicted the next *Face Look*, *B* = 0.11, *t*(108) = 6.98, *p* < 0.001 (the target look), confirming the temporal dependency effect. The lag 2 *Face Look* also predicted the target *Face Look*, *B* = 0.05, *t*(108) = 3.52, *p* = 0.001. The *Face Look* final model accounted for 3.46% of the variance in the empty model (percent of variance accounted for, PVAF)^24^ (see Table 2 for final model parameters and Table S1 for the expanded equation). The relationship between observed and predicted *Face Look* durations is described in Fig. 2A.

**Table 2:** Predictors of *Face Look* Durations in the FFSF Final Model.

*Fixed Effects*	*β*	*SE*	*t*	*df*	*p*
Intercept (*B*_00_)	0.26	0.02	17.08	108	<0.001
Look Duration One Previous (*B*_10_)	0.11	0.02	6.98	108	<0.001
Look Duration Two Previous (*B*_20_)	0.05	0.01	3.52	108	0.001
***Random Effects***	***Variance***		***χ*** ^**2**^	***df***	***p***
Intercept(*r*_0i_)	0.02		608.57	108	<0.001
Look Duration One Previous (*r*_1i_)	0.01		122.70	108	0.16
Look Duration Two Previous (*r*_2i_)	0.01		93.82	108	>0.50
Residual Variance (*ε*_*ij*_)	0.19		—	—	—

*Note*: Fixed effects in this table represent the estimated mean effect in the overall sample. Random effects represent the estimated variation between individual infants. Residual variance represents variance unaccounted for in the model.

In previous models other effects of interest were tested and not retained in the final model (see Table S1 for a summary of model building). There was no effect of episode for Still-Face vs. Face-to-Face and Reunion, *B* = −0.02, *t*(108) = −1.56, *p* = 0.12, or for Face-to-Face vs. Reunion, *B* = −0.02, *t*(108) = −1.70, *p* = 0.09. There was no interaction between temporal dependency (lag 1) and episode effects for Still-Face vs. Face-to-Face and Reunion, *B* = 0.01, *t*(108) = 0.48, *p* = 0.63, or for Face-to-Face vs. Reunion, *B* = 0.01, *t*(108) = 0.66, *p* = 0.51. There was no significant effect of the previous *Away Look* on the target *Face Look*, *B* = −0.01, *t*(108) = −0.54, *p* = 0.65.

#### Away Looks

The final model for predicting look durations away from the parent was one in which one previous look duration (*D*_*n−1 ij*_), the effect of the Still-Face compared to the Face-to-Face and Reunion (*SF vs. FF & RE*_*ij*_), their interaction (SF vs. FF & RE * D_*n−1 ij*_), and the intercept of *Away Looks* (*π*_*0i*_) predicted the next *Away Look* duration (*Y*_*ij*_), as described in this simplified model of level 1 effects:

$${Y}_{ij}={\pi }_{0i}+{\pi }_{1i}{D}_{n-1ij}+{\pi }_{2i}SF\,vs.\,FF\,\& \,R{E}_{ij}+{\pi }_{3i}SF\,vs.\,FF\,\& \,RE\ast {D}_{n-{1}_{ij}}+{\varepsilon }_{ij}$$

Specifically, one previous *Away Look* (lag 1) predicted the next *Away Look*, *B* = 0.05, *t*(108) = 2.94, *p* < 0.01 (the target look), confirming the temporal dependency effect. There was an effect of episode for Still-Face vs. Face-to-Face and Reunion, *B* = 0.21, *t*(108) = 9.23, *p* < 0.001, such that *Away Looks* were longer in the Still-Face than the Face-to-Face and Reunion. There was an interaction between temporal dependency (lag 1) and episode effects. Temporal dependency in the Still-Face episode was lower than the Face-to-Face and Reunion episodes, *B* = −0.10, *t*(108) = −4.10, *p* < 0.001, but there was no difference between the Face-to-Face and Reunion episodes, *B* = −0.02, *t*(108) = −0.90, *p* = 0.37. That is, when accounting for the main effect of Still-Face vs. Face-to-Face and Reunion and their interaction, the effect of previous *Away Look* was attenuated in the Still-Face. The *Away Look* final model accounted for 11.11% of the variance in the empty model (PVAF)^24^ (see Table 3 for final model parameters and Table S2 for the expanded equation). The relationship between observed and predicted *Away Look* durations is described in Fig. 2B.

**Table 3:** Predictors of *Away Look* from Parent Durations in the FFSF Final Model.

Fixed Effects	β	SE	t	df	p
Intercept (*B*_00_)	0.46	0.02	18.85	108	<0.001
Look Duration One Previous (*B*_10_)	0.05	0.02	2.94	108	<0.01
Still-Face vs. Face-to-Face and Reunion (*B*_20_)	0.21	0.02	9.23	108	<0.001
Look Duration One Previous * Still-Face vs. Face-to-Face and Reunion Interaction (*B*_30_)	−0.10	0.02	−4.10	108	<0.001
**Random Effects**	**Variance**		**χ** ^**2**^	**df**	**p**
Intercept (*r*_0i_)	0.23		611.41	103	<0.001
Look Duration One Previous (*r*_1i_)	0.01		134.10	103	0.02
Still-Face vs. Face-to-Face and Reunion (*r*_2i_)	0.03		204.99	103	<0.001
Look Duration One Previous * Still-Face vs. Face-to-Face and Reunion Interaction (*r*_3i_)	0.01		106.49	103	0.39
Residual Variance (*ε*_*ij*_)	0.36		—	—	—

*Note*: Fixed effects in this table represent the estimated mean effect in the overall sample. Random effects represent the estimated variation between individual infants. Residual variance represents variance unaccounted for in the model.

In previous models that were not retained the other effects of interest were tested (see Table S2 for a summary of model building). There was no effect of episode for Face-to-Face vs. Reunion, *B* = −0.03, *t*(108) = −1.89, *p* = 0.06. The lag 2 *Away Look* did not predict the target Away Look, *B* = 0.01, *t*(108) = 0.63, *p* = 0.53. There was no effect of the previous *Face Look* on the target *Away Look*, *B* = 0.01, *t*(108) = 0.23, *p* = 0.82.

#### Face Looks and Away Looks

In both *Face Look* and *Away Look* models of individual look durations, *Face Looks* and *Away Looks* did not predict one another. To assess whether the temporal dependency of *Face Look* and *Away Look* were consistent at the level of *infants*, we examined the correlation of temporal dependency terms from each final model. Pearson’s correlation quantified the association of the estimated slope values for *Face Look* and *Away Look* temporal dependency across all episodes of the FFSF, controlling for all other final model terms. Infants’ level of temporal dependency in *Face Looks* was associated with infants’ level of temporal dependency in *Away Looks*, *r*(107) = 0.25, *p* < 0.01.

## Discussion

In studies of infant interaction^25^, looks toward a social partner index engagement and looks away from the partner serve to regulate arousal associated with social engagement. The current findings provide evidence of an underlying structure, temporal dependency, which structures both engagement- and disengagement-linked looks. There were nevertheless differences in how these two types of looks were affected by a break in parental interaction. Temporal dependency in the duration of looks to the parent in the FFSF was unaffected by a break in parental interaction (the Still-Face)—suggesting infants were responsible for structuring their own social engagement. However, temporal dependency in the duration of *Away Looks* from the parent in the FFSF was attenuated when the parent adopted a still face, suggesting that an absence of parental interaction decreased infants’ structuring of their *disengagement*.

### Temporal Dependency in the FFSF

The current study evidenced temporal dependency in both *Face Look* and *Away Look* durations during interaction. One *Face Look* duration predicted the next *Face Look* duration, and one *Away Look* duration predicted the next *Away Look* duration. In sum, infant look durations during interaction do not follow a random Poisson process^26^. The structuring of looking behavior at the level of individual looks (rather than the overall sum of look durations or the overall frequency of looks) is crucial to understanding how infant social engagement and disengagement are structured in time.

During the course of interaction, the durations of *Face Looks* and *Away Looks* were independent of one another. The duration of a given *Face Look* was not predicted by how long the infant had previously looked away from the parent. In parallel, the duration of a given *Away Look* from the parent was not predicted by how long the infant had previously looked at the parent. The independence of *Face Look* durations and *Away Look* durations provides further evidence that these two forms of attention function independently in time^5^.

By contrast, the analysis of each infant’s *individual level* of temporal dependency revealed that levels of *Face Look* and *Away Look* temporal dependency were associated. This suggests that infants exhibit relatively stable levels of temporal dependency to social and nonsocial targets. The degree to which temporal dependency represents a stable individual difference characteristic across other stimuli is an open research question. Earlier, we found evidence for the generality of temporal dependency, finding that previous look durations toward a non-contingent social stimulus predict subsequent look durations in a habituation protocol^4^. However, this research did not directly address either social interaction with the parent or looks away from the social target. The current results indicate that the temporal duration of looks at the social target are unaffected by a break in parental interaction, while the temporal duration of looks away from the parent declines when parents cease interacting.

### Parental Influence on *Away Look* Temporal Dependency

The Still-Face is a potent experimental disruptor of interaction in which the parent is asked to not respond to the infant^1,2^. The current findings suggest that the consistency of the durations of looks at a social partner is unaffected by the Still-Face. By contrast, the consistency of the durations of looks away from the parent *was* affected by the Still-Face—specifically, temporal dependency was weaker during this pause in interaction. This contrast suggests that infant temporal structuring of engagement during social looking is not dependent on parental interaction, while the disruption of interaction affects infants’ structuring of their own non-social engagement.

During the Still-Face the overall duration of infant gazing at the parent declines relative to episodes of interaction, and looking away from the parent increases^2^. Moreover, there is evidence for a logarithmic decline in gazing at the parent during the course of the Still-Face^27^. However, there has been little understanding of the temporal structure of the looks that constitute these patterns. In the context of prior investigations, the current findings highlight the role of interaction with the parent in providing temporal structure to infants’ looks away from the parent. They underline the necessity of considering both looks *to* the social partner and looks *away* from the social partner, which appear to reflect different temporal processes.

### Limitations

The generality of the present study’s findings are limited by several factors. First, infant looks away from the parent’s face were used to index social disengagement. However, the precise target of the infants’ *Away Looks* were not coded. Future research on the temporal dependency of looks to nonsocial targets could clarify similarities and differences in infant attention to the social and nonsocial world. Second, the FFSF is an experimental manipulation of parental behavior. However, we did not code specific parental behaviors in the current study. Therefore, inferences about the influence of specific parental behaviors on infant temporal dependency cannot be made. Future research might employ a dyadic analysis approach to more fully investigate the role of the parent in their infant’s temporal dependency during interaction. Notably, previous work did not find an effect of maternal behavior on either infant gaze duration or temporal dependency^5^. Nevertheless, the role of parental behaviors in maintaining the temporal structuring of patterns of infant disengagement is a propitious topic of future research. Despite limitations, the current findings indicate that infants structure their own behavior in time, but that social interaction has a differential impact on the sequencing of specific behaviors.

### Conclusion

Temporal dependency supplements well-established looking measures by accounting for non-random variance at the level of individual looks. By more fully characterizing looking behavior, temporal dependency is providing insights into how infants structure their attention and engagement. As temporal dependency is found across multiple contexts^4,5^, it will aid in the prediction of the duration of individual behaviors as they occur in time. This prediction is crucial to modeling looking behaviors analytically, in both software simulations and robotic prototypes^28–31^. Ultimately, the results contribute to a growing understanding of the role of temporal processes (memory) in structuring behavior across actors and contexts^3,32^.

## Methods

### Participants

The current sample of participants was drawn from three major metropolitan areas and diverse in their race/ethnicity. All experimental protocols were approved by the University of Miami Institutional Review Board and were carried out in accordance with their guidelines for human subjects research. Informed consent was obtained from participants’ legal guardian at the time of study enrollment. A summary of sample demographic characteristics is presented in Table 4. A total of 109 parents and their six-month-old infants completed the FFSF. This sample includes 54 infants examined in a previous report^27^. This report did not include analyses of temporal dependency or gazes away from the parent. Infants had no sensory or motor impairments that impeded completion of activities, or identified metabolic, genetic, or progressive neurological disorders. All infants had a gestational age between 37 and 41 weeks and a birth weight of at least 2500 grams. Participants were drawn from a larger longitudinal study of infants at high and low familial risk for autism spectrum disorder (low-risk *n* = 43, high-risk *n* = 65). At 36 months of age, 93 participants completed diagnostic evaluations, and 16 participants were diagnosed with autism spectrum disorder. To ensure that subsequent diagnosis did not influence any effects of interest, a model was constructed which tested whether diagnostic category affected look-level variables. There were no significant effects for model parameters on the basis of diagnostic category, and all participants were included in analyses for the current study (see Supplemental Materials for details).

**Table 4:** Demographic Characteristics.

Racial/Ethnic Group	
Hispanic	24 (22.02%)
Non-Hispanic White	70 (64.22%)
African American	4 (3.67%)
Asian/Unknown/Other	11 (10.09%)
Age in Months	6.11 (*SD* = 0.34)
Female	44 (40.37%)

### Measures

Temporal dependency was examined during the FFSF protocol. The parent was seated facing their infant and instructed to play with their child as they would at home for three minutes (Face-to-Face), to stop interacting and adopt a still face for two minutes (Still-Face), then to begin interacting again for three minutes (Reunion). These durations were selected to maximize sampling of interaction dynamics (see Tronick *et al*.^1^) and to assess infant reaction to a period of parental non-responsiveness sufficient to elicit the still-face effect^2^. The entire procedure was video-recorded with cameras facing the infant’s and the parent’s faces, as well as an overall view of both partners. Within the FFSF an infant’s *Face Look* was defined as a period of visual fixation at the parent’s face. An *Away Look* was defined as a period of visual fixation away from the parent’s face (to any non-target area of the environment). There was no minimum look duration imposed, other than the technical limit of video frames, i.e., no looks less than 33.337 ms. Infant *Face Look* and *Away Look* durations were reliably coded by trained experts (*ICC* = 0.83, *SD* = 0.06, 25% of infants).

### Statistical Modeling

We used a multi-level modeling approach in which successive looks (level 1) were nested within infants (level 2). Using an iterative approach, fixed effects (mean effects across subjects) were maintained in a model if (a) their inclusion significantly improved model fit and (b) their coefficients were significant. Because multi-level models specifically afford inclusion of between-person variance in effects, we paired fixed and random effects for all variables to simplify modeling. That is, all models included random effects for all individual-level predictors regardless of significance. Differences between model deviances were used to assess model fit. Difference values of model deviances follow a (central) chi square distribution with the degrees of freedom equal to the difference in degrees of freedom. Fixed effects were unstandardized to allow for interpretation of associations in raw units.

The same steps were used to model both *Face Look* and *Away Look* durations. We began with a model that included an intercept and between-subjects differences in that intercept (differences in each infant’s mean look duration) to confirm that multi-level modeling was appropriate and to establish a baseline model for model fit testing. Variables were added based on the central hypotheses and the variables needed to test these hypotheses. We began with the previous look duration (lag 1) to test for temporal dependency. We then added episode effects (Face-to-Face and Reunion vs. Still-Face, Face-to-Face vs. Reunion) to confirm that typical changes in look durations was present. Then we added the look duration two previous (lag 2) to check if there was temporal dependency two lags previous, as an exploration of the extent of temporal dependency. With the basic assumptions established, we then modeled research question 1 for Face Look and question 2 for *Away Look* by adding the interaction between the previous look duration (lag 1) and the two episode terms. The final model was selected when all variables had been tested and either retained or eliminated based on fixed effect significance and model improvement. With a final model established we then tested if the look-level element of research question 3 by adding the previous other look type (e.g., for *Face Look* the previous *Away Look*). See Tables S1 and S2 and supplementary materials for model building details. Parameter estimates were then extracted for each individual from each final model to answer the individual-level look element of research question 3.

## Electronic supplementary material

Supplementary Materials

Supplementary Data

## Data Availability

All data generated or analyzed during this study are included in this published article (and its Supplementary Information files).
