# Supplementary material for: A break in parental interaction does not affect the temporal dependency of infant social engagement, but disrupts non-social engagement
Source: Sci Rep. 2018 Oct 11;8:15150. doi: 10.1038/s41598-018-33270-9 (PMC6181912; doi:10.1038/s41598-018-33270-9)
Supplement: Supplementary file 1 — Supplementary Materials [file 41598_2018_33270_MOESM1_ESM.pdf]

A break in parental interaction does not affect the temporal dependency of infant social engagement, but disrupts non-social engagement

Supplementary Information

Whitney I. Mattson,[1] Daniel S. Messinger,[2,3,4,5] Devon N. Gangi,[6] Nicholas D. Myers[7,8]

[1]*Center for Biobehavioral Health, The Research Institute at Nationwide Children's Hospital, Columbus, Ohio, United States of America*
[2]*Department of Psychology, University of Miami, Coral Gables, Florida*
[3]*Department of Pediatrics, University of Miami, Coral Gables, Florida, United States of America*
[4]*Department of Music Engineering, University of Miami, Coral Gables, Florida, United States of America*
[5]*Department of Electrical & Computer Engineering, University of Miami, Coral Gables, Florida, United States of America*
[6]*MIND Institute, University of California, Davis, Sacramento, California, United States of America*
[7]*Department of Counseling, Educational Psychology and Special Education, Michigan State University, East Lansing, Michigan, United States of America*
[8]*Department of Kinesiology, Michigan State University, East Lansing, Michigan, United States of America*

Supplementary Information

When examining the influence of autism spectrum disorder status on temporal dependency models, two parallel models were constructed for *Face Look* and *Away Look*. Each incorporated a dummy-coded predictor of autism spectrum disorder status for each of the fixed effects in the final model. For *Face Look*, children with and without later autism spectrum disorder did not differ in temporal dependency one look back, B = -0.02, $t(91)$ = -0.33, p =.74, two looks back, B = -0.01, $t(91)$ = -0.21, p =.84, or in their mean look duration, B = 0.08, $t(91)$ = 1.90, p =.06. For *Look Away*, children with and without later autism spectrum disorder did not differ in temporal dependency one look back, B = 0.03, $t(91)$ = 0.62, p = .54, two looks back, B = 0.05, $t(91)$ = 1.09, p =.28, between the still-face and face-to-face/reunion, B = 0.07, $t(91)$ = 1.24, p = .22, and mean look duration, B = .02, $t(91)$ = 0.32, p = .75.

Summary of Face Look modeling

The FFSF *Face Look* model was constructed by first generating a base or empty model (Model 1), using the mean look and random differences between infants in that mean look. This model showed non-trivial dependency between individuals, *ICC* = .83; that is, infants differed from each other in their look durations, rationalizing a multi-level modeling analysis approach. Next, a model was constructed including both the previous look duration as a predictor and random differences between infants in the strength of that prediction (Model 2), which was a significant improvement over the empty model, χ^2 (3, J = 109) = 245.27, p < .001. Next, a model was constructed which added the effect of the FFSF protocol, with two dichotomous variables encapsulating the differences between the Still-Face episode and episodes with interaction (Face-to-Face and Reunion), and the differences between interaction before (Face-to-Face) and after (Reunion) perturbation of the interaction (Model 3). This model also included terms for random differences between infants in the predictive strength of each episode-based prediction. Model 3 was a significant improvement over Model 2, χ^2 (9, J = 109) = 31.08, p < .001. The next model added a second previous look duration as a predictor, as well as random differences

between infants in that prediction (Model 4). This was a significant improvement over Model 3, χ^2 (6, *J* = 109) = 166.43, *p* < .001. However, neither of the two episode effect dichotomous variables were significant in this model, β_{30} = .01, SE (β) = .01, *t* (108) = 1.23, *p* = .22, β_{60} = -.04, SE (β) = .02, *t* (108) = -1.75, *p* = .09. Consequently, the final model (Model 5) dropped both episode effect variables. Model 5 was a significant improvement over Model 2, χ^2 (4, *J* = 109) = 169.38, *p* < .001. While Model 4 fit better than Model 5, χ^2 (11, *J* = 109) = 28.13, *p* < .001, all fixed effects were significant in Model 5. A model incorporating two interaction terms was also tested (Model 6), one between the previous look duration and the Still-Face contrast, and the other with the previous look duration and the Face-to-Face vs. Reunion contrast. However, Model 6 was not a significant improvement over Model 5, χ^2 (26, *J* = 109) = 33.68, *p* = .14, and neither interaction term was significant. An alternate model was also tested which assessed whether the current *Face Look* duration was predicted by the previous *Look Away* (Model 7). While this model was an overall improvement over Model 5, χ^2 (18, *J* = 109) = 168.28, *p* < .001, the previous *Look Away* duration, β_{30} = .01, SE (β) = .01, *t* (108) = 1.23, *p* = .22, was not significant.

The final model (Model 5) accounted for 3.46% of the variance in the empty model (PVAF; [24] In this final model, the previous look duration and the look duration two previous were predictive of a given look duration. Temporal dependency was indicated in the FFSF for *Face Look* durations, β_{10} = .11, SE (β) = .02, *t* (108) = 6.98, *p* < .001. The results are summarized in Table 3; the prediction of *Face Look* durations is depicted in Figure 2.

In summary, the equation for the final model of Face Look was:

$$Y_{ij} = \beta_{00} + \beta_{10}D_{n-1\,ij} + \beta_{20}D_{n-2\,ij} + (\varepsilon_{ij} + r_{0i} + r_{1i}D_{n-1\,ij} + r_{2i}D_{n-2\,ij} + r_{0i} * r_{1i}D_{n-1\,ij} + r_{0i} * r_{2i}D_{n-2\,ij} + r_{1i}D_{n-1\,ij} * r_{2i}D_{n-2\,ij})$$

Where Y_{ij} represents the duration in seconds of the *j*th in a series of looks for infant *i*. β_{00}represents the mean intercept of *Face Look* durations in seconds, and r_{0i} represents random

differences between infants from that mean. β_{10} is the mean change in *Face Look* durations for every one second change in the previous *Face Look* duration, and r_{1i} represents random differences between infants in that change. β_{20} is the mean slope of change in *Face Look* durations for every one second change in the Face Look duration two previous, and r_{2i} represents random differences between infants in that change. β_{10} and β_{20} index temporal dependency. ε_{ij} is unexplained residual variance in for the *j*th *Face Look* duration for the *i*th infant. Additionally, the covariances of all random effect terms (r_{0i} - r_{3i}) are included in this model

Summary of Away Look modeling

The FFSF *Away Look* model was constructed by first generating a base or empty model (Model 1), using the mean look and random differences between infants in that mean look. This model showed non-trivial dependency between individuals, *ICC* = .88, the rationale for using a multi-level modeling approach. Next, a model was constructed including both previous look duration as a predictor and random differences between infants in the strength of that prediction (Model 2), which was a significant improvement over Model 1, χ^2 (3, J = 109) = 441.28, p < .001. Next, a model was constructed which added the effect of the FFSF protocol, with two dichotomous variables encapsulating the differences between the Still-Face episode and episodes with interaction (Face-to-Face and Reunion), and the differences between interaction before (Face-to-Face) and after (Reunion) perturbation of the interaction (Model 3). These also included terms for the random differences between infants in the predictive strength of each episode-based prediction. This was a significant improvement over Model 2, χ^2 (4, J = 109) = 241.21, p < .001. Next, we constructed a model which added a second previous *Away Look* duration as a predictor, as well as random differences between infants in that prediction (Model 4). This was a significant improvement over Model 3, χ^2 (6, J = 109) = 224.82, p < .001. A model incorporating two interaction terms was next tested, one between the previous look duration and the Still-Face contrast, and the other with the previous look duration and the Face-to-Face vs.

Reunion contrast (Model 5). This model was a significant improvement over Model 4, χ^2 (15, *J* = 109) = 31.48, *p* < .01; however, only the interaction term between previous *Away Look* duration and the Still-Face contrast was significant. Next a model was constructed which included two previous *Away Look* durations, the two episode variables, and the interaction between previous *Away Look* duration and the Still-Face contrast (Model 6). This model was a significant improvement over Model 4, χ^2 (7, *J* = 109) = 20.63, *p* < .01, however, the *Away Look* two previous term was no longer significant, β_{20} = .01, SE (β) = .01, *t* (108) = 0.63, *p* = .53. As this term was not required to model interactions, it was dropped from the next model (Model 7). Model 7 included one previous *Away Look* duration, the two episode variables, and the interaction between previous *Away Look* duration and the Still-Face contrast. Model 7 was a significant improvement over the previous nested model, Model 3, χ^2 (6, *J* = 109) = 24.00, *p* < .001. In Model 7 the dichotomous variable contrasting the Face-to-Face and Reunion episodes was non-significant, β_{30} = -.03, SE (β) = .01, *t* (108) = -1.89, *p* = .06. This term was dropped in the final model (Model 8), which included one previous *Away Look* duration, the Still-Face contrast, and the interaction between previous *Away Look* duration and the Still-Face contrast. Model 8 was a significant improvement over the previously nested model, Model 2, χ^2 (6, *J* = 109) = 24.00, *p* < .001, and all fixed effects were significant in Model 8. An alternate model was also tested, assessing whether the current *Away Look* duration was predicted by the previous *Face Look* duration—including all predictors in the final model (Model 8). While this alternate model (Model 9) was an improvement in fit over the final model (Model 8), χ^2 (7, *J* = 109) = 244.77, *p* < .001, the fixed effect of previous *Face Look*, was non-significant, β_{30} = .01, SE (β) = .03, *t* (108) = 0.23, *p* = .82.

The *Away Look* final model (Model 8) accounted for 11.11% of the variance in the empty model (PVAF; [24] In the final model, the *Away Look* duration one previous, the Still-Face contrast, and their interaction term were significant. The *Away Look* durations were longer in the Still-Face compared to the Face-to-Face and the Reunion. During the Still-Face episode, there

was a smaller effect of temporal dependency compared to the Face-to-Face and Reunion episodes. Temporal dependency was found in the FFSF for successive looks away, β_{10} = .05, SE (β) = .02, t (108) = 2.94, p < .01. The results are summarized in Table 3; the prediction of *Away Look* durations is depicted in Figure 3.

In summary, the equation for the final model of Away Look was:

$$Y_{ij} = \beta_{00} + \beta_{10}D_{n-1\,ij} + \beta_{20}SF\ vs.FF\ \&\ RE_{ij} + \beta_{30}SF\ vs.FF\ \&\ RE_{ij} * D_{n-1_{ij}} + (\varepsilon_{ij} + r_{0i} + r_{1i}D_{n-1\,ij} + r_{2i}SF\ vs.FF\ \&\ RE_{ij} + r_{3i}SF\ vs.FF\ \&\ RE * D_{n-1_{ij}} + r_{0i} * r_{1i}D_{n-1\,ij} + r_{0i} * r_{2i}SF\ vs.FF\ \&\ RE_{ij} + r_{0i} * r_{3i}SF\ vs.FF\ \&\ RE * D_{n-1_{ij}} + r_{1i}D_{n-1\,ij} * r_{2i}SF\ vs.FF\ \&\ RE_{ij} + r_{1i}D_{n-1\,ij} * r_{3i}SF\ vs.RE * D_{n-1_{ij}} + r_{2i}SF\ vs.FF\ \&\ RE_{ij} * r_{3i}SF\ vs.FF\ \&\ RE * D_{n-1_{ij}})$$

Where Y_{ij} represents the duration in seconds of successive Away Looks, the *j*th in a series of looks for infant *i*. β_{00} represents the intercept of *Away Look* durations, r_{0i} represents random differences between infants from that intercept. β_{10} is the slope of change in *Away Look* durations for every one second change in the previous *Away Look* duration, r_{1i} represents random differences between infants in that change. β_{10} indexes temporal dependency. β_{20} is the slope of change in *Away Look* durations due to parental Still-Face relative to the Face-to-Face and Reunion, r_{2i} represents random differences between infants in that change. β_{30} is the slope of change in *Away Look* durations for every one second change in the previous *Away Look* duration during parental Still-Face relative to the Face-to-Face and Reunion, r_{3i} represents random differences between infants in that change. ε_{ij} is unexplained residual variance in for the *j*th *Away Look* duration for the *i*th infant. Additionally, the covariances of all random effect terms (r_{0i} - r_{3i}) are included in this model.

Table S1. *Summary of FFSF Face Look Model Building*

Model	σ^2	τ_{00}	χ^2 *vs. Previous Model*	*df*	*p*
1. Empty Model	.1948	.0215	-	-	-
2. Look Duration One Previous and its Random Effect	.1896	.0210	245.27	3	< .001
3. Look Duration One Previous and Episode Contrasts and their Random Effects	.1869	.0199	31.08	9	< .001
4. Look Duration One Previous, Two Previous, Episode Contrasts and their Random Effects	.1858	.0198	166.43	6	< .001
5. Look Duration One Previous, Two Previous, and their Random Effects (*Final Model*)	.1884	.0201	169.38	4	< .001
6. Look Duration One Previous, Two Previous, Episode Contrasts, the Interaction of Look Duration One Previous and the Episode Contrasts, and their Random Effects	.1851	.0198	33.68	26	.14
7. Look Duration One Previous, Two Previous, *Away Look* One Previous, and their Random Effects	.1865	.0204	38.33	5	< .001

Note: The Final Model equation was: $Y_{ij} = \beta_{00} + \beta_{10}D_{n-1\,ij} + \beta_{20}D_{n-2\,ij} + (\varepsilon_{ij} + r_{0i} +$

$r_{1i}D_{n-1\ ij} + r_{2i}D_{n-2\ ij} + r_{0i} * r_{1i}D_{n-1\ ij} + r_{0i} * r_{2i}D_{n-2\ ij} + r_{1i}D_{n-1\ ij} * r_{2i}D_{n-2\ ij})$

Table S2. *Summary of FFSF Away Look Model Building*

Model	σ^2	τ_{00}	χ^2 *vs. Previous Model*	*df*	*p*
1. Empty Model	.3966	.0693	-	-	-
2. Look Duration One Previous and its Random Effect	.3764	.0702	441.28	3	< .001
3. Look Duration One Previous, Episode Contrasts, and their Random Effects	.3544	.0597	213.74	9	< .001
4. Look Duration One Previous, Two Previous, Episode Contrasts, and their Random Effects	.3544	.0073	224.82	6	< .001
5. Look Duration One Previous, Two Previous, Episode Contrasts, the Interaction of Look Duration One Previous and the Episode Contrasts, and their Random Effects	.3505	.0560	31.48	15	< .01
6. Look Duration One Previous, Two Previous, Episode Contrasts, the Interaction of Look Duration One Previous and the Still-Face Contrast, and their Random Effects (vs. Model 4)	.3521	.0557	20.63	7	< .01
7. Look Duration One Previous,	.3519	.0556	24.00	6	< .001

Episode Contrasts, the Interaction of Look Duration One Previous and the Still-Face Contrast, and their Random Effects (vs. Model 3)					
8. Look Duration One Previous, Still-Face Contrast, the Interaction of Look Duration One Previous and the Still-Face Contrast, and their Random Effects (*Final Model* vs. Model 2)	.3592	.0549	197.39	9	< .001
9. Look Duration One Previous, *Face Look* One Previous, Still-Face Contrast, the Interaction of Look Duration One Previous and the Still-Face Contrast, and their Random Effects	.3544	.0544	244.77	7	< .001

Note: The Final Model equation was:

$$Y_{ij} = \beta_{00} + \beta_{10} D_{n-1\,ij} + \beta_{20} SF\ vs.FF\ \&\ RE_{ij} + \beta_{30} SF\ vs.FF\ \&\ RE_{ij} * D_{n-1_{ij}} + (\varepsilon_{ij} + r_{0i} + r_{1i} D_{n-1\,ij} + r_{2i} SF\ vs.FF\ \&\ RE_{ij} + r_{3i} SF\ vs.FF\ \&\ RE * D_{n-1_{ij}} + r_{0i} * r_{1i} D_{n-1\,ij} + r_{0i} * r_{2i} SF\ vs.FF\ \&\ RE_{ij} + r_{0i} * r_{3i} SF\ vs.FF\ \&\ RE * D_{n-1_{ij}} + r_{1i} D_{n-1\,ij} * r_{2i} SF\ vs.FF\ \&\ RE_{ij} + r_{1i} D_{n-1\,ij} * r_{3i} SF\ vs.RE * D_{n-1_{ij}} + r_{2i} SF\ vs.FF\ \&\ RE_{ij} * r_{3i} SF\ vs.FF\ \&\ RE * D_{n-1_{ij}})$$

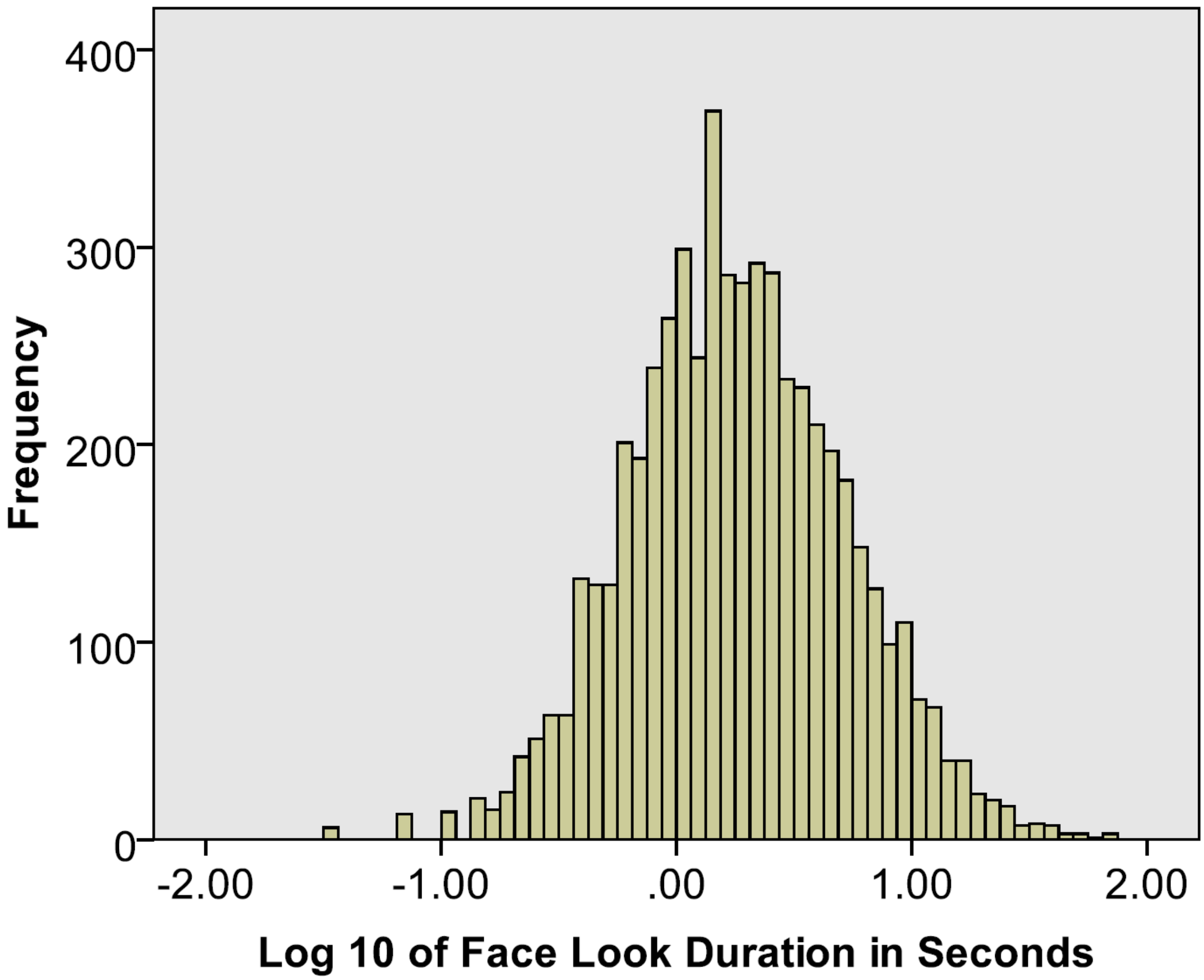

Figure S1. A histogram of the distribution of *Face Look* durations following a Log10(x+1) transformation.

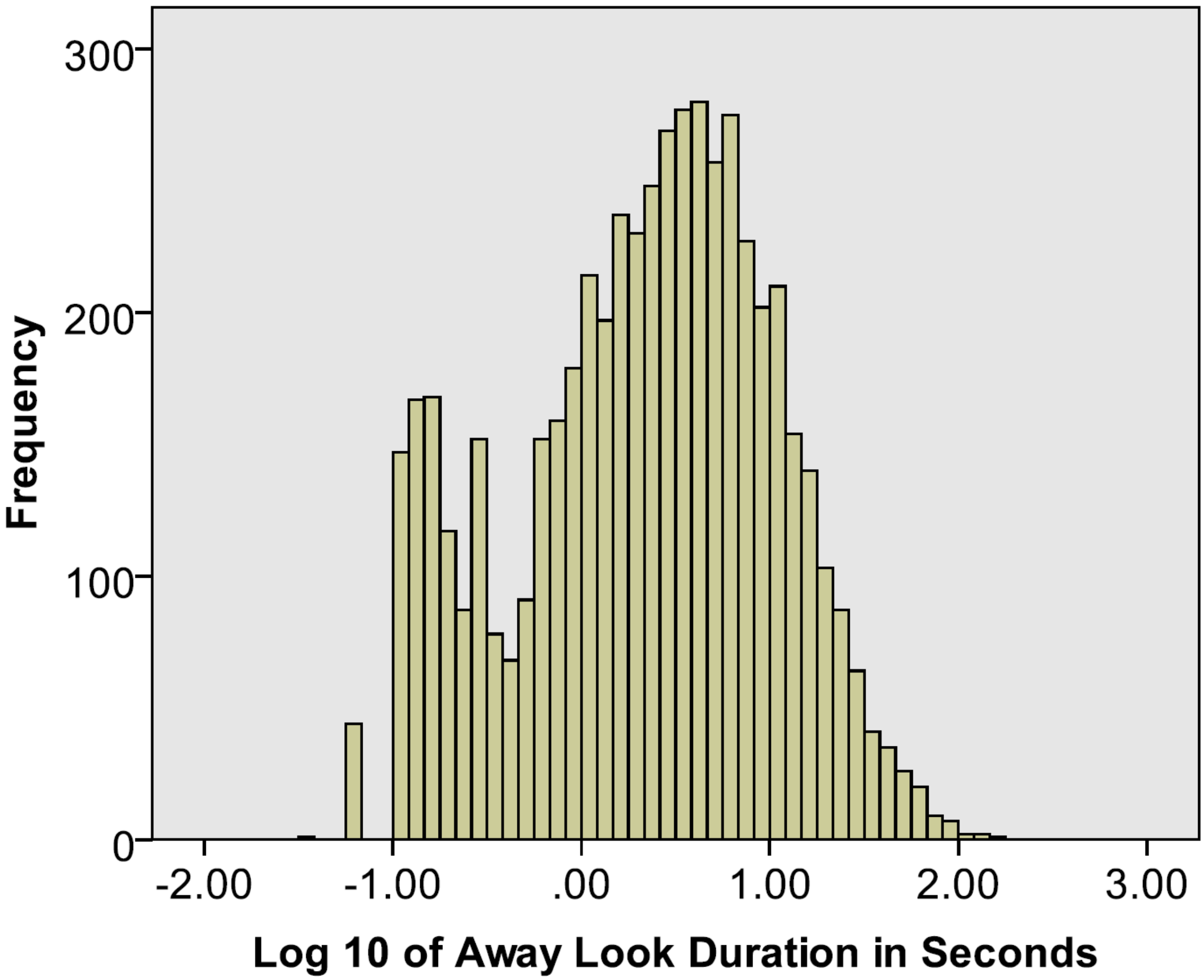

Figure S2. A histogram of the distribution of *Away Look* durations, following a Log10(x+1) transformation.
